# Mesoscopic Simulation of Core–Shell Composite Powder Materials by Selective Laser Melting

**DOI:** 10.3390/ma16217005

**Published:** 2023-11-01

**Authors:** Tao Bao, Yuanqiang Tan, Yangli Xu

**Affiliations:** 1 Institute of Manufacturing Engineering, Huaqiao University, Xiamen 361021, China; bao15713660965@163.com (T.B.); ylxu@hqu.edu.cn (Y.X.); 2 National & Local Joint Engineering Research Center for Intelligent Manufacturing Technology of Brittle Material Products, Huaqiao University, Xiamen 361021, China

**Keywords:** selective laser melting, core–shell composite powder materials, melt pool dynamics, enhanced particle distribution

## Abstract

Mechanical ball milling is used to produce multi-materials for selective laser melting (SLM). However, since different powders have different particle size distributions and densities there is particle segregation in the powder bed, which affects the mechanical properties of the printed part. Core–shell composite powder materials are created and used in the SLM process to solve this issue. Core–shell composite powder materials selective laser melting (CS-SLM) has advanced recently, expanding the range of additive manufacturing applications. Heat storage effects and heat transfer hysteresis in the SLM process are made by the different thermophysical characteristics of the core and the shell material. Meanwhile, the presence of melt flow and migration of unmelted particles in the interaction between unmelted particles and melt complicates the CS-SLM molding process. It is still challenging to investigate the physical mechanisms of CS-SLM through direct experimental observation of the process. In this study, a mesoscopic melt-pool dynamics model for simulating the single-track CS-SLM process is developed. The melting characteristics of nickel-coated tungsten carbide composite powder (WC@Ni) were investigated. It is shown that the powder with a smaller particle size is more likely to form a melt pool, which increases the temperature in the area around it. The impact of process parameters on the size of the melt pool and the distribution of the reinforced particles in the melt pool was investigated. The size of the melt pool is significantly affected more by changes in laser power than by changes in scanning speed. The appropriate control of the laser power or scanning speed can prevent enhanced particle aggregation. This model is capable of simulating CS-SLM with any number of layers and enables a better understanding of the CS-SLM process.

## 1. Introduction

Having both the high strength and stiffness of the reinforcing phase and the ductility and toughness of the metal matrix, metal matrix composites (MMCs) have outstanding mechanical characteristics at high temperatures, strong wear resistance, and great chemical stability [1]. They are therefore often employed in areas requiring high-end engineering, such as those in the medical and aerospace industries, where they must withstand high loads [2], severe wear [3] and tear [4], and high temperatures [5].

As it overcomes the constraints of product shape, additive manufacturing (AM) has sparked substantial studies on AM in several domains. Since adding reinforcing phases may increase a material’s overall qualities including strength [6], hardness [7], wear resistance and elastic modulus [8], the fabrication of particle-reinforced metal matrix composites (PRMMCs) applying AM also attracts a lot of interest. Additionally, the issues with machinability and malleability related to traditional PRMMCs manufacturing can be reduced with AM [6,9]. A better synergistic impact results from the combination of AM technology with MMCs.

Currently, combining ceramic and metal powders is the first step in the selective laser melting (SLM) synthesis of particle-reinforced metal matrix composites. Through DEM of the spreading in the SLM process, Yao et al. discovered that the particle size and distribution of the powder are closely related to the segregation behavior of the powder bed and that smaller particle sizes exacerbate the phenomenon [10]. Wei et al. discovered that the particle segregation that resulted from insufficient powder mixing also worsened the chemical heterogeneity in the powder. These led to a problem regarding uneven powder distribution immediately during the stage of substance preparation [11]. Ge et al. studied the TiC/Ti6Al4V SLM method and discovered that as the TiC concentration grew, more laser interactions occurred with the powder, amplifying the phenomenon of multiple laser reflections in the powder bed. As a result, the melt pool’s maximum temperature rose along with the powder bed’s overall rate of absorption [12]. During the SLM process, the stability of the melt pool will be affected by the uneven distribution of the powder, which will lead to the development of inclusion-related defects such as balling, porosity, and cracking. Dudina et al. suggested surface modification of reinforcing particles and metal cladding on their outer layers to create composite powder materials with a core–shell structure to reduce the problem of uniformity of distribution between different powders [13]. Through the process of chemical vapor deposition (CVD), Davydova et al. coated about 2 μm of metallic cobalt (Co) on the outside layer of 5–35 μm boron carbide (B4C) ceramic particles [14]. It demonstrates that SLM can create metal-coated ceramic particles with a core–shell structure for simple geometries. Using fluidized bed chemical vapor deposition (FBCVD) together with a chemical plating process, Zhang et al. produced WC-Co composite powders with a core–shell structure, and the powders showed excellent printability in SLM [15].

The previous studies proved the use of core–shell composite powder materials in PRMMCs by SLM, and they additionally looked at the microstructure as well as mechanical properties after manufacturing. The movement and distribution of the reinforcing particles during the SLM molding process, as well as the molten pool temperature history, are all directly related to the product’s mechanical properties. However, it can be challenging to carry out real-time measurements of the temperature history of the molten pool and the movement of the reinforced particles due to the complexity of the SLM processes.

Numerical simulations are often utilized to study laser-material interactions on multiple scales in addition to experimental approaches, revealing the evolution of the melt pool [16], the mechanisms of defect development [17], and other phenomena in the SLM process [16,18]. Ao et al. developed a transient model of the Ti6Al4V SLM process that includes the impacts of surface tension, the Marangoni effect, and vapor recoil [19]. The model studies the mechanism governing the evolution of the melt pool as well as the processes of heat transfer, fluid flow, melting and solidification during the SLM process. For the simulation research on multichannel and multilayer processes in LPBF processes concerning the void issues of lack of fusion in LPBF processes, Bayat et al. developed a multiphysics field model [20].

It was found that the lower layers exhibit a lack of fusion defects since the thermal energy of the fluid in the lower laminar flow is usually less than in the upper layers. Adopting the VOF approach, Xu et al. followed the molten pool’s free liquid surface to discover the effect of surface laser power and scanning speed on the flow and surface morphology [21]. At the same time, research on the melt pool’s center temperature showed that it changed significantly due to the melt’s unstable motion, which was caused by heat conduction and accumulated heat.

There are examples of numerical models for multi-material SLM being effective in addition to the numerical simulations for single-material SLM mentioned above. TiC/AlSi10Mg [22], AlN/AlSi10Mg [23], and TiC/Ti6Al4V [24] composite powders’ melt pool dynamics and laser absorptivity were studied during SLM by Dai and Gu et al. Understanding the thermodynamic and dynamical characteristics of the melt and reinforcing particles in the melt pool is made possible by visualizing the flow field.

Also, it may reveal how mixed powder materials absorb laser energy in the SLM process. An SLM mesoscopic model for mixed powders was put out by Sun et al. based on the VOF. In718/Cu10Sn mixed powders at various volume ratios were studied using the model to comprehensively describe heat transfer, melt flow, and melt-pool shape [25]. To simulate multi-material, multi-trajectory, and multi-layer SLM processes for 316L and Cu10Sn powders, Gu et al. established an integrated modeling framework that involves multi-material powder deposition and laser powder interactions [26]. Through the model, the dynamics of the melt pools at different levels as well as the thermal history and melt-pool evolution of two powder materials with various thermophysical characteristics were studied.

However, mixed powders are the primary focus of all efforts for multi-material mesoscopic simulations. To the best of our knowledge, no research has been done on the mesoscopic simulation of multi-material SLM with core–shell composite powder materials.

Under the flow of thermal fluid in VOF multiphase flow, a multiphase mesoscopic molten-pool dynamics model is developed in this study for the SLM process of WC@Ni composite powder. The model includes consideration of thermal convection, heat transfer, thermal radiation, Marangoni convection and vapor recoil. The model was applied to simulate the single-track CS-SLM, and data on the melt evolution process and the melt temperature history were obtained. The effects of various process parameters on the melt-pool temperature, existence time and size, and reinforcing particle dispersion in the melt pool were studied by comparing the simulation results under various process settings.

## 2. Modeling Framework and Methodology

### 2.1. Modeling Framework

**Figure 1:**
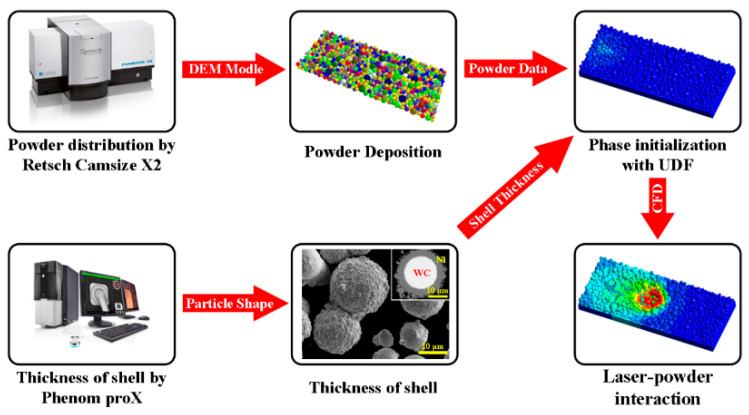
Framework for CS-SLM modeling.

The CS-SLM multiphase mesoscopic melt-pool dynamics model’s computational framework is depicted in Figure 1. As demonstrated, a particle size analyzer (Retsch Camsize X2, Retsch Technology Inc., Hahn, Germany) first examines the composite powder’s size distribution. Based on these results, the discrete element method (DEM) generates the particle distribution and initializes the powder deposition in the scanning layer. Meanwhile, SEM (PhenomproX, SEM purchases from phenom-world BV in the Netherlands. The company is located in Eindhoven, The Netherlands) is used to assess the composite powders’ exterior thickness. After that, the powder data is transferred to a CFD model, where laser interaction with powders is realized, along with calculations for heat and mass transport, melting and solidification, gravitational force, surface tension force, recoil pressure, and the heat source with Gaussian distribution. The single-track CS-SLM’s molten-pool development, heat transfer properties and reinforced-particle movement are all simulated using the model.

### 2.2. Basic Assumptions

With the SLM mesoscopic modeling of WC@Ni composite powders, the following assumptions were made:

1. Assume that the viscous, incompressible thermally conductive fluid melt convection is laminar [25];
2. Enthalpy-porosity modeling of material melting and solidification processes takes place [26];
3. Evaporation-related quality of material loss is not taken into consideration [27];
4. Ignore the impact of protective gas flow on the melt pool’s pattern of flow;
5. Regarding particle radius, the core–shell powder’s outer metal layer’s thickness remains constant.

### 2.3. Governing Equations

Based on the assumptions, the mass conservation equation, the momentum conservation equation, and the energy conservation equation are the governing equations describing the SLM heat and mass transfer of WC@Ni composite powders [28].

Mass conservation equation

(1) $$\frac{\partial \rho }{\partial t}+\nabla \cdot \left(\rho V\right)=0$$

Momentum conservation equation

(2) $$\frac{\partial }{\partial t}(\rho V)+\nabla \cdot \left(\rho VV\right)=-\nabla P+\nabla \cdot \mu (\nabla V+\nabla V^{T})+p_{b}+p_{s}$$

Energy conservation equation

(3) $$\frac{\partial }{\partial t}\left(\rho H\right)+\nabla \cdot \left(\rho VH\right)=\nabla \cdot \left(k\nabla T\right)+q_{pt}+q_{l}+q_{loss}$$

The surface morphology of the molten pool is traced utilizing the volume of finite fluid (VOF) approach [29]. The advection equation, which represents the gas when *V_F_* = 0 and the fluid phase when *V_F_* = 1, is what determines the development of the VOF method14 [30]. The VOF equation is expressed as follows:

(4) $$\frac{\partial V_{F}}{\partial t}+\nabla \cdot \left(\vec{v}V_{F}\right)=0$$

### 2.4. Heat Source

The laser beam’s power is assumed to be Gaussian-distributed, and to simplify calculations, only consider the thermal effects of the laser beam’s reflection. The following equations are utilized with a rotating body heat source in this research [31]:

(5) $$q=\frac{9PA}{R^{2}\pi H\left(1-\frac{1}{e^{3}}\right)}\exp\left(\frac{-9\left(x^{2}+y^{2}\right)}{R^{2}\log\left(\frac{H}{z}\right)}\right)$$

where *P* is the laser power, *A* is the laser absorption rate of the materials, *R* is the laser radius and *H* is the penetrated depth of the heat source.

### 2.5. Boundary Conditions

**Figure 2:**
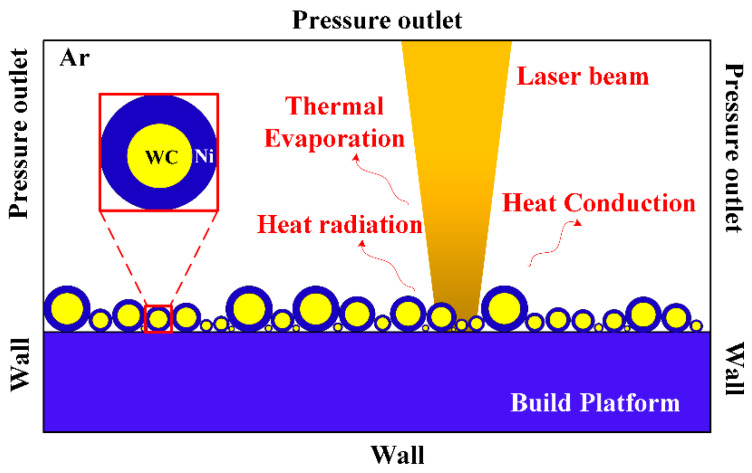
Schematic of the simulation model.

The schematic boundary conditions of the simulation model employed in this research are shown in Figure 2. While the sides and top of the powder layer are given as pressure outlets, the sides and bottom of the substrate serve as walls. Heat convection, heat radiation, and evaporation are the primary methods used to lose heat in the substrate and powder layers.

(6) $$q_{loss}=q_{conv}+q_{rad}+q_{evap}$$

(7) $$q_{conv}=h_{c}\left(T-T_{ref}\right)$$

(8) $$q_{rad}=k\varepsilon \left(T^{4}-T_{ref}^{4}\right)$$

(9) $$q_{evap}=0.54\left[P_{a}\exp\left(\frac{\Delta H_{lv}}{R_{v}T_{boil}}\left(1-\frac{T_{boil}}{T}\right)\right)\right]$$

where *h_c_* is the thermal conductivity, *T_ref_* is the environmental temperature, *k* and *ε* are the Boltzmann constant and radiation intensity, respectively. In evaporative heat loss, *P_a_* is the atmospheric pressure. ∆*H_lv_* is the effective evaporation enthalpy. *R_v_* is the gas constant. *T_boil_* is metal evaporation temperature.

Surface forces resulting from surface tension (including Marangoni shear) and recoil pressure resulting from metal evaporation from the melt pool’s surface are the driving forces for flow in the melt pool and are controlled by the equations:

(10) $$F_{\gamma }=\gamma Kn+\left(\nabla T-\nabla T\left(\nabla T\cdot n\right)\right)\frac{\partial \gamma }{\partial T}$$

where *γ*, *K* and **n** stand for the free surface normal, curvature, and tension at temperature *T*, respectively.

(11) $$P_{recoil}=0.54P_{a}\exp\left[\frac{\Delta H_{lv}}{R_{v}T_{boil}}\left(1-\frac{T_{boil}}{T}\right)\right]$$

Additionally, the laser heat source, metal evaporation heat loss, and metal evaporation-induced recoil pressure in the model are loaded by the UDF program.

### 2.6. Properties of Material of WC@Ni Composite Powder

This model comprises the metallic phase Ni, the reinforcing phase WC, and the gas phase argon. Table 1 lists the physical properties of the materials used for the simulation, including the material’s density, thermal conductivity, specific heat capacity, and viscosity.

### 2.7. Numerical Method

**Figure 3:**
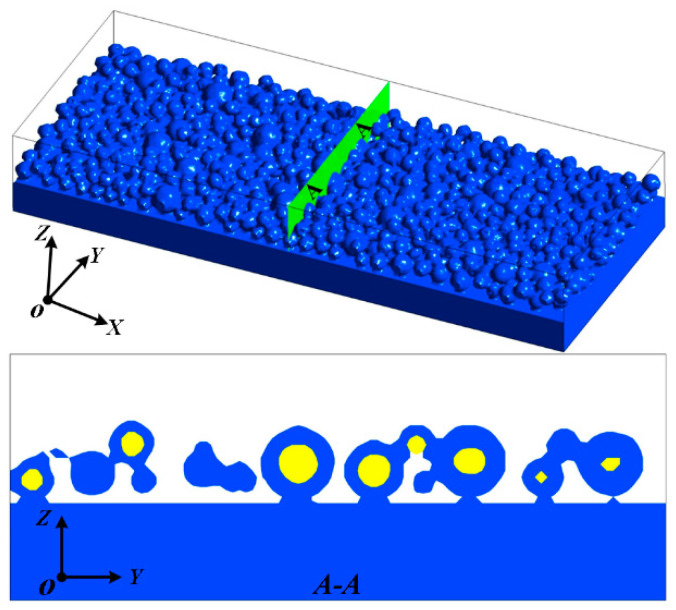
Initialized powder bed for WC@Ni Composite Powder and cross-section.

A powder bed model is used to simulate the interaction between the laser and the powder, which reproduces the surface shape as the melt pool evolves while responding accurately to the complex state of the powder layer. This study initially develops a powder model applying DEM software (Rocky 4.3.3) by the actual particle-size powder condition of the powder and inputs the powder information into the model. The results of the simulation produced in this study’s initialization are shown in Figure 3. Transient computations are used to simulate the SLM process. The numerical fluid dynamics model is utilized in this work with the Fluent program to solve all the equations. For numerical simulations, accuracy is critical and depends on the type of cell, mesh size, and heat source employed for the powder bed. The model has a hexahedral mesh since the powder melt and melt pool are crucial components. With a regular grid spacing of 4 μm and a dimension of 100 × 400 × 360 μm^3^, the computational domain has 950,000 cells. The PRESTO! implicit discretization of the continuity and energy conservation equations is employed. The SIMPLE solver is used and the time step is set to 2 × 10^−8^ s. The calculation was run on the high-performance computer platform at the University of Huaqiao.

## 3. Experiment Setup

**Figure 4:**
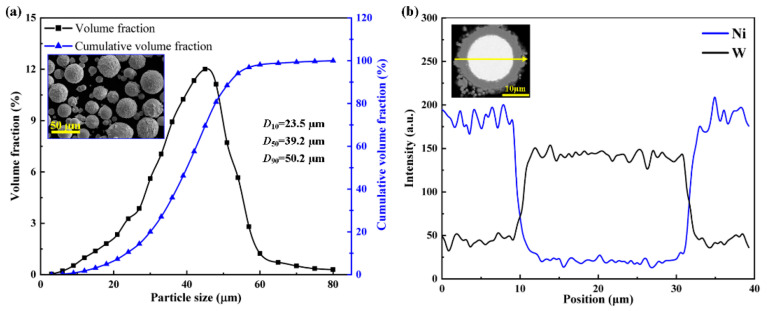
WC@Ni composite powder’s particle size distribution and morphology (**a**) and SEM with a WC@Ni particle with EDS elemental mapping of Ni and W (**b**).

The Institute of Materials and Processing, Guangdong Academy of Sciences provided the WC@Ni (nickel-coated tungsten carbide) composite powder used in this study. The composite powder consists of a core layer made of tungsten carbide and an outer layer made of nickel metal that has excellent sphericity. A Dynamic Particle Size and Shape Analyzer (Camsizer X2, Hahn, Germany) is used to measure the specifics of the powder size distribution (D10 = 23.5 μm, D50 = 39.2 μm, and D90 = 50.2 μm). Figure 4a illustrates the WC@Ni composite powder’s particle size distribution, its shape under SEM, and a cross-section of a single particle. The distribution of Ni and W elements in the composite powder is evident from the EDS map. The powder material needs to be dry before the experiment to avoid porosity and agglomerations carried on by powder flow during the SLM process.

Utilizing SLM 125 (SOLUTION, Frankfurt, Germany), the SLM experiment of WC@Ni composite powders is accomplished. A 400 W fiber laser (Nd-YAG-400, Frankfurt, Germany) with a wavelength of 1.064 μm is installed in the equipment. The spot diameter of the laser is 70 μm, and the laser energy distribution is Gaussian. Argon-filled protective gas is used for the SLM experiment on WC@Ni composite powder. Single-track scanning experiments on WC@Ni composite powders are performed using various processing parameters, as shown in Table 2, to verify the simulation results.

## 4. Results and Discussion

### 4.1. Experiment Verification

**Figure 5:**
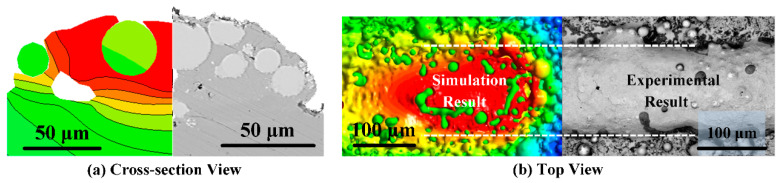
The single-track scanning (P = 140 W, V = 600 mm/s) cross-section view (**a**) and top view (**b**) of simulation results compared to the experiment.

The reliability of the simulation results depends heavily on the agreement of the results with the experimental findings. WC@Ni composite powder melt-pool morphology simulation and experimental comparison results are displayed in Figure 5. The simulation results are in greater agreement with the test results, according to a comparison of the simulation and test of the molten pool cross-section and top view. Table 3 shows a comparison of the simulation and experimental results for the characteristic molten pool dimensions for various process parameters. Melt-pool depth and width errors can only be 10% at most. This shows the numerical model used in this work can predict the shape of a molten pool.

### 4.2. Molten Pool Dynamics of Melting WC@Ni Composites Powders

**Figure 6:**
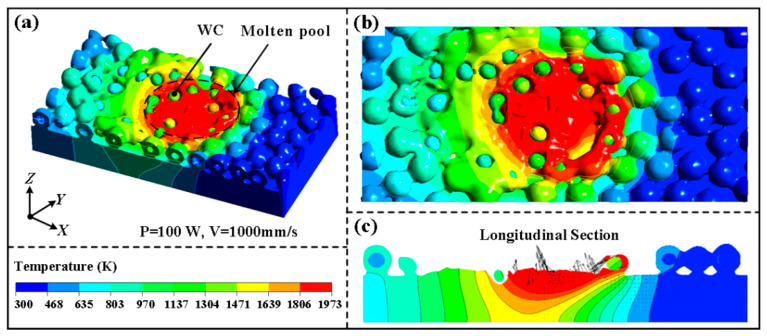
Temperature distribution (**a**): top view (**b**), temperature and velocity distribution of the longitudinal section (**c**) (P = 100 W, V = 1000 mm/s).

During the SLM process, the temperature distribution and melt-pool velocity vectors are shown in Figure 6. The Marangoni shear brought on the surface tension and the recoil pressure produced by metal evaporation perpendicular to the melt indication are the key factors driving the melt flow. It is evident from the temperature distribution in Figure 6b that the melt pool’s front and center have a higher temperature and a higher temperature gradient, but the area around them has a more uniform temperature distribution. According to the velocity vector in Figure 6c, the flow rate is highest close to the front of the melt pool, which is consistent with the area with the largest temperature change. Therefore, sudden changes in physical properties are what produces the violent flow at the melt-pool front, but surface tension and recoil pressure are what generates the flow at the melt-pool end.

**Figure 7:**
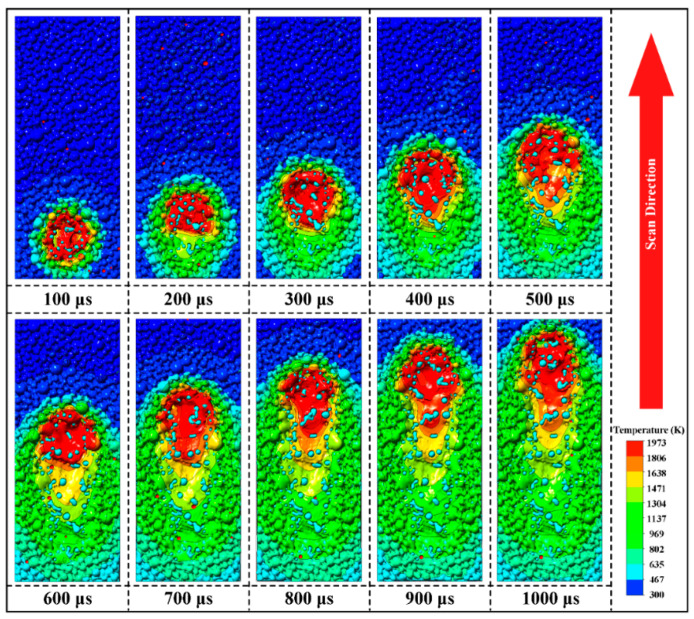
Morphology evolution, temperature and reinforced particle distribution during single track on WC@Ni composite powder bed (P = 140 W, V = 600 mm/s).

Figure 7 displays the evolution of the molten pool morphology, temperature field and distribution of reinforcing particles during SLM of WC@Ni composite powders. The laser beam’s initial position is at (0.1, 0, 0), and it is moving along the X-axis. The molten pool formed of the outside Ni of the composite powder can be seen traveling along with the laser. Simultaneously the interior WC particles that have not melted in the composite powder are gradually exposed. Due to the salient difference between the thermophysical properties of metallic Ni and WC particles, an unsmooth melt-pool isotherm forms. The temperature in the red region around the isotherm is greater than the temperature of the liquid-phase line of the Ni (1973 K), yet the melting point of WC is much higher (3314 K), as indicated by the light-colored spheres. Thus, although the WC particles in this area stay solid, the Ni can melt. The unmelted WC particles in the melt are driven by the molten Ni. The flow rate of the liquid metal steadily reduces as the melting temperature decreases, and it finally stops flowing after solidification. This enables the position of WC particles in the melt pool to be determined, producing the occurrence when an inlay forms on a molten pool’s surface. This result is consistent with Zhang’s simulation of W particles embedded in the surface of the molten pool during EBM of mixed Cu and tungsten powders [35].

### 4.3. Temperature Evolution of the Molten Pool

**Figure 8:**
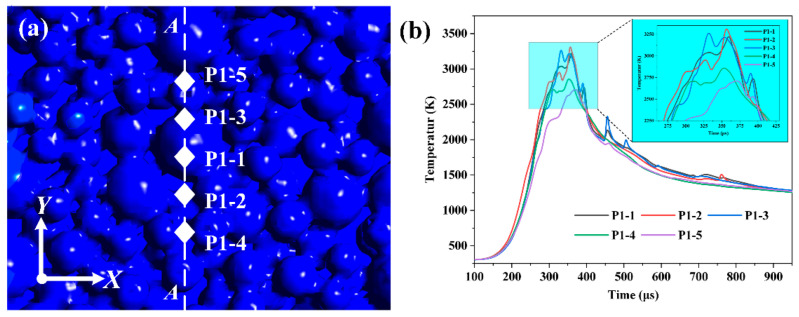
The schematic distribution of the profiles in the powder bed (**a**) and temperature development history of the five points (**b**).

According to previous research, the complex thermodynamics occurring in the melt pool during the SLM process affect how the reinforcing particles migrate and distribute. This study analyzes temperature history to analyze the SLM process thermodynamics. As shown in Figure 8a, five monitoring locations are set up on the horizontal plane to monitor the temperature history of the powder bed. P1-1, P1-2, P1-3, P1-4 and P1-5 are distributed apart by 0.05 mm from the substrate’s top surface.

Their coordinates are P1-1 (0.3, 0, −0.05), P1-2 (0.3, 0.05, −0.05), P1-3 (0.3, 0.05, −0.05), P1-4 (0.3, −0.10, −0.05), and P1-5 (0.3, 0.10, −0.0). Under the SLM process situations of 140 W and 800 mm/s, Figure 8b exhibits the temperature development history at five monitor locations. By comparing the temperature histories of the monitor points, it is clear that the temperature maximum increases with closeness to X = 0 and that it is greater in the positive direction of the Y-axis than in the opposite way. The melt’s development along the Y-axis is the primary factor leading to this phenomenon. Under the same situations, a melt pool can form simply in the negative direction of the Y-axis since there are smaller particles, while the contrary is harder to achieve. Heat convection and heat conduction between materials cause the temperature history curves of close monitoring points to repeatedly cross over. This causes the monitoring points’ temperature curves to finally coincide.

**Figure 9:**
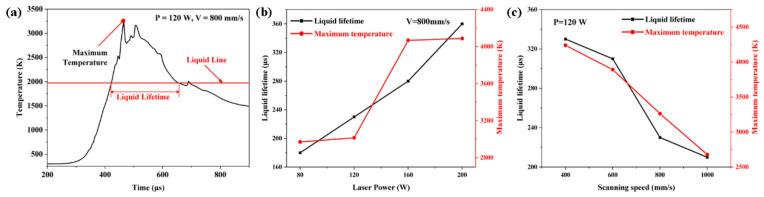
The calculated temperature profile (P = 120 W, V = 800 mm/s) at point P1 (**a**), liquid lifetime and maximum temperature at the scan track using various (**b**) laser powers (V = 800 mm/s) and (**c**) scanning speeds (P = 120 W).

At 140 W, 800 mm/s process conditions, Figure 9a shows the temperature curve of the model’s monitoring point. The temperature profile shows both the maximum temperature in the molten pool and the liquid lifetime. Figure 9b,c show, for various laser power and scanning speeds, respectively, the maximum temperature in the melt pool and the liquid lifetime. When the laser power increases, the molten pool’s maximum temperature and liquid lifetime both increase. The molten pool’s maximum temperature at the specific scanning speed was 2971 K, and it existed for 180 μs when the laser power was 80 W. When the laser’s power increased to 200 W, the molten pool’s maximum temperature increased to 4088 K, and the liquid lifetime extended to 360 μs. At a specific laser power, the melt pool’s maximum temperature was 4240 K, its scanning speed was 400 mm/s, and the liquid lifetime was 330 μs. The melt pool’s maximum temperature declined to 2679 K and the time it spent existing decreased to 210 μs, however, when the scanning speed was increased to 1000 mm/s.

### 4.4. Influence of Process Parameters on Molten Pool Morphology

**Figure 10:**
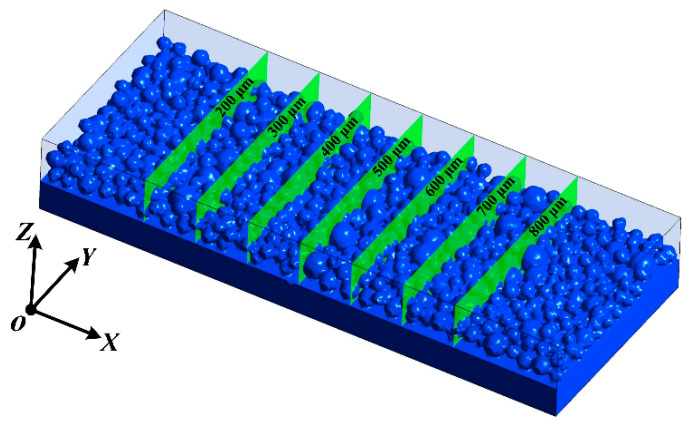
The illustration of the locations of the extracted cross-sections.

Multiple sections of the model are set up to record the melt-pool dimensions under various process parameters, which are statistically evaluated to study the impact of process factors on melt-pool dimensions. Seven planes are established using YOZ as the datum, one plane per 100 m, as illustrated in Figure 10.

**Figure 11:**
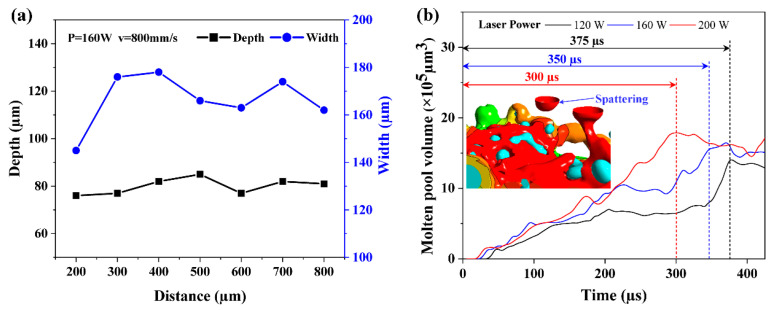
Depth and width of a single-track (P = 160 W, V = 800 mm/s) melt pool at various cross-sections on WC@Ni composite powder bed (**a**). The single-track melt-pool volume development history (V = 800 mm/s) with varying laser power (**b**).

Figure 11a illustrates the relationship between the depth and width of the molten pool and the distance between the coordinate origins under the process circumstances of laser power 160 W and scanning speed 800 mm/s. Melt-pool depth and breadth are indicated by black squares and blue circles, respectively. As can be observed from the extraction findings, the accumulated effect of heat leads the depth and width of the melt pool to start stabilizing after a period of laser–powder interaction. At the same scanning speed and different laser power, Figure 11b exhibits the melt-pool volume development history. After experiencing a rapid increase, the melt-pool volume eventually stabilizes. With increased laser power, this experience time shortens. After 300 μs, the melt-pool volume increase slows down and even decreases at a laser power of 200 W. When the melt temperature is above the vaporization line of the Ni, the evaporated metal vapor will generate a recoil pressure perpendicular to the melt surface. At this point, the melt flow rate increases and the melt moves in all directions while the melt pool forms a downward concave shape. In severe cases, this will cause the melt to break away from the powder bed, resulting in the phenomenon of splashing, as shown in Figure 11b. This causes a decrease in melt volume, which seriously affects the stability of the melt pool. The melt-pool volume increase slows down at 120 W after 375 μs, due to the low energy density and the laser’s ability to adequately offset other energy loss including heat conduction and heat radiation. As the laser energy density increases, the formation of the molten pool relies mainly on Marangoni convection. The volume of the molten pool at this stage is relatively stable. As the laser energy density is further increased, the molten pool exists for a longer lifetime and can flow more fully, as shown in Figure 11a. The melt volume will gradually converge to a stable condition at a laser power of 160 W after 350 μs when the laser radiation energy and the energy lost by heat conduction and thermal radiation can reach a generally stable balance.

**Figure 12:**
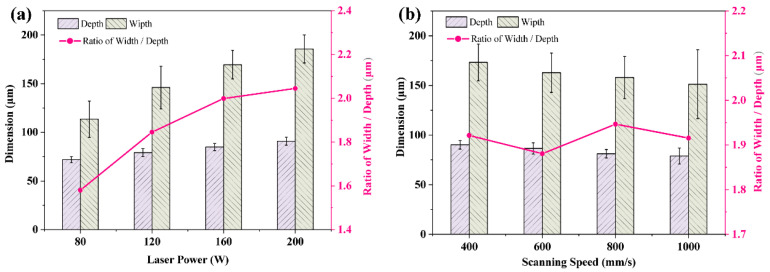
The depth, width and width-to-depth ratio of melt-pool dimensions change with different laser powers (**a**) and scanning speeds (**b**).

Figure 12a,b present, for various laser strengths and scanning rates, respectively, the statistical results of the melt-pool dimensions. When the scanning speed is determined, the melt pool’s depth and width increase with improving laser power, and its width-to-depth ratio also dramatically rises. As the laser power improved from 80 W to 200 W, the melt-pool width increased from 113.5 μm to 185.75 μm and the melt-pool depth increased from 71.83 μm to 90.83 μm, as shown in Figure 12a. When the laser power is determined, the melt pool’s depth and width decrease as the scanning speed increases, but the width-to-depth ratio does not vary much. As the scanning speed rose from 400 mm/s to 1000 mm/s, the melt pool width dropped from 173.25 μm to 151.17 μm and the melt pool depth decreased from 90.17 μm to 78.97 μm, as illustrated in Figure 12b. Statistics on the molten pool’s cross-sectional dimensions suggest the change in laser power had a greater impact on those dimensions than the scanning speed. This is mainly due to the fact that the temperature in the melt pool is proportional to the power and inversely proportional to one-half of the power of the scanning speed [36].

### 4.5. Influence of Process Parameters on Reinforced-Particle Distribution

**Figure 13:**
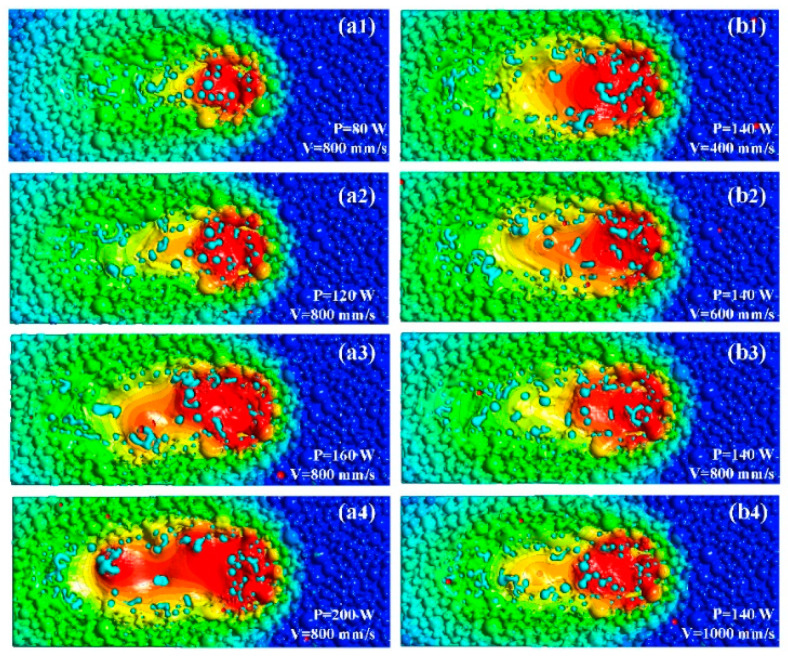
The reinforced-particle distribution in the molten pool at various laser powers (**a1**–**a4**) and scanning speeds (**b1**–**b4**). Light coloured spheres indicate unmolten WC particles. The red part indicates the molten metal pool.

A series of simulations were conducted for a single track using laser powers of 80 W, 120 W, 160 W, and 200 W and scanning speeds of 400 mm/s, 600 mm/s, 800 mm/s, and 1000 mm/s to study the effects of process parameters on the distribution of reinforced particles in the molten pool. Figure 13 shows the molten pool’s surface morphology and the dispersion of reinforced particles. The agglomeration of the enhanced particles increases with the laser energy density. This conclusion is consistent with that reached by Dai in modeling the AlN/AlSi10Mg SLM process [23]. The findings demonstrate that when laser power increases, more reinforced particles are spread on both sides of the molten pool. The increased particles in the center of the molten pool greatly decrease when the laser power arrives at 160 W or 200 W, but the number of particles on each side increases, as seen in Figure 13(a3,a4). The number of reinforced particles aggregating in the melt pool’s center grows as the scanning speed rises. As shown in Figure 13(b3,b4), the number of particles in the melt pool’s center greatly increases when the scanning speed arrives at 800 mm/s or 1000 mm/s. Comparing the simulation results with various process parameters, the reinforced particles migrate to the center of the melt pool as the scanning speed increases while reinforced particles gather at the sides of the melt pool as the laser power increases.

## 5. Conclusions

To investigate the thermal-fluid behavior of WC@Ni composite powder in the SLM process, a multi-phase mesoscopic melt-pool dynamics model was developed.

Initial powder beds containing Ni and reinforced particle WC were created, and the effects of the melt-pool dynamics, temperature evolution process, solidification trajectory, and reinforcement particle distribution were studied during the SLM process.

The conclusions are as follows:

1. The material’s physical properties at its front location heavily vary with the melt pool’s evolution, causing it to flow irregularly. However, the flow behind the melt pool is driven by surface tension brought on by temperature and pressure perpendicular to the melt surface carried on by metal evaporation.
2. During the SLM process, WC@Ni composite powders have a complicated variation in temperature. In the powder bed, smaller particle sizes are more likely to generate melt pools, which enhance the temperature. However, heat transfer and heat convection, among particles, eventually cause the temperature at various spots in the powder bed to concord.
3. The melt-pool size is greatly influenced by the laser power and scanning speed. The melt-pool dimensions are more sensitive to variations in laser power, according to statistical analysis of the cross-section dimensions of the melt pool in the simulation results.
4. The reinforced particles distributed in the melt pool depend on the laser power and scanning speed. The simulation results for various process parameters show that increasing laser power or reducing scanning speed will increase the probability of the enhanced particles migrating to the center of the melt pool.

## Figures and Tables

**Table 1:** Physical properties of WC@Ni Composite Powder.

Symbol	Parameter	Unit	Ni [32]	WC [33]	Ar [34]
*ρ_s_*	Solidus Density	kg∙m^−3^	8500	15,630	-
*ρ_l_*	Liquidus Density	kg∙m^−3^	7900	-	-
*ρ_g_*	Gaseous Density	kg∙m^−3^	-		1.784
*T_s_*	Liquidus temperature	K	1726	3058	-
*T_l_*	Solidus temperature	K	1973	3413	-
*λ_s_*	Thermal conductivity of solid	W∙m^−1^∙K^−1^	75	110	-
*λ_l_*	Thermal conductivity of liquid	W∙m^−1^∙K^−1^	62		-
*λ_g_*	Thermal conductivity of gas	W∙m^−1^∙K^−1^	-		0.017
*C_ps_*	Specific heat of solid	J∙kg^−1^∙K^−1^	515	292	-
*C_pl_*	Specific heat of liquid	J∙kg^−1^∙K^−1^	595	-	-
*C_pl_*	Specific heat of gas	J∙kg^−1^∙K^−1^	-	-	520.64
*μ*	Viscosity of metal	kg∙m^−1^∙s^−1^	3.68 × 10^−3^	-	0.0005
*L_m_*	Latent heat of melting	J∙kg^−1^	2.93 × 10^5^	5.6 × 10^5^	-
*σ*	Surface tension	kg∙s^−2^	1.778	-	-
*dσ*/*dT*	Temperature of surface tension	kg∙s^−2^∙K^−2^	−3.4 × 10^−3^	-	-
*R*	Universal gas constant	J∙mol^−1^∙K^−1^	8.314	8.314	-
*η*	Laser beam absorptivity	-	0.4	0.82	-

**Table 2:** The processing parameters of the SLM.

Parameter	Value	Unit
The radius of the laser spot	70	μm
Layer Thicknesses	60	μm
Laser Power	80, 120, 140, 160, 200	W
Scanning Speed	400, 600, 800, 1000	mm/s

**Table 3:** Comparing the experimental dimensions and calculated characteristic values of the single-track melt pool.

	Laser Power	Scanning Speed	Simulation		Experiment		Relative Error	
			Width	Depth	Width	Depth	Width	Depth
Case 1	140 W	400 mm/s	173.25	90.17	159.25	99.75	8.08%	9.60%
Case 2	140 W	600 mm/s	162.83	86.58	150.25	93.63	7.73%	7.53%
Case 3	200 W	800 mm/s	185.75	90.83	197.1	96.63	6.11%	6.00%

## Data Availability

The data presented in this study are available on request from the corresponding author.
